# An Essay on the Philosophy of Medical Science

**Published:** 1845-05

**Authors:** 


### REVIEWS.

---

ART. V.—*An Essay on the Philosophy of Medical Science.* By ELISHA BARTLETT, M.D., Professor of the Theory and Practice of Medicine, in the University of Maryland. Philadelphia: Lea & Blanchard. 1844. 8vo. pp. 312.

There are some things about which men may differ. There are some things to which they may attach too much importance. There are some things for which a man may endeavor to create an interest. But the healing art is not one of those things.

Men may differ as to the best form of government; or whether mankind would not be better off in a state of nature, as they suppose, without government. Jurists may differ, and do differ, as to the best civil code. But the time never will come when mankind will differ about the value of the healing art. Here even doctors do not differ. We may question in a general sense, we allow, whether more men are killed or cured by drugs; but yet, we repeat again, the time never will come, while man is in the state in which he now is, when the good physician will not be looked upon as the good christian; as a man above price, a light set upon a hill, which cannot be hid.

It is not as many suppose, that physicians of olden time were distinguished, and had their names handed down to us, because they were scholars, and were celebrated for their attainments in literature, and other accomplishments: not so. They immortalized themselves by healing the sick; by doing good. Physicians, in every age, have made an impression upon the world, and that impression will never be effaced.

From the cradle, nay, even from the womb to the grave, the doctor's services are felt and demanded.

Philanthropy applies more appropriately to the medical profession than to any other class of mankind.

Medical schools may multiply, the standard of the profession may be lowered, never so much; quacks may vend their nostrums with a diploma in their hands; but there stands the science, firm as the pillars of the earth; and her true disciple, the good physician, like the mariners compass, no discovery will ever supercede him.

Medicine is as truly a science as any other department of knowledge to which the term is applied. No science is more varied or extensive. The nature of this science, and in what it consists, we may dispute about. That a correct understanding of its principles is all important none will deny.

It is with these feelings and impressions that we undertake to review the work before us.

La Place, in the introduction to his Celestial Mechanics, expresses some concern lest intelligent astronomers might have some difficulty in understanding his principles. So vague and indefinite have been the notions of medical men as to what constitutes medical science, and so unlike is this Essay to any work which has preceded it, that we fear, by a large majority of readers it will neither be appreciated nor understood.

Various are the reasons which will induce the different classes of medical men to decry this work. Already have we heard, and that too as a matter of congratulatory remark among some teachers of medicine, that the book fell still-born from the press.

All systematists will feel a deep interest in its rejection. The disciples of Cullen, Brown, Broussais, &c., will agree upon this, if upon nothing else. They will be unwilling to give up their theories, especially as the author of the new doctrine does not propose a theory of his own. *The classification and arrangements of the phenomena and their relationships,* will be to them a poor substitute for *inductive*

*reasoning;* and but a meagre definition of science and philosophy.

All medical science, says Dr. Bartlett, consists in ascertained facts, or phenomena, or events; with their relations to other facts, or phenomena, or events; the whole classified and arranged.

We read this proposition, with those which immediately follow it, to a physician of ordinary intelligence, who called at our office, and taking up the book, asked us to state to him, in a few words, in what Dr. Bratlett made the science of medicine to consist. He forthwith exclaimed, *there is no philosophy in that.* Such we candidly believe will be the judgment passed upon this work by a majority of its readers.

It is our deliberate opinion that the doctrine of our author will not be comprehended by a majority of physicians. They will be so unwilling to recieve it that they will not take the trouble to study it, so as to take hold of the true meaning of the author. For although no man could express his thoughts in a more clear and intelligible manner than the author has done, yet when the reader comes to inquire, what does this new doctrine amount to, if he feels the force of the reasoning, he will be so completely unsettled, that he will be like a boy lost in his father's forest: he may know the trees individually, but will be unable to find his way out of the woods.

The doctrine of Dr. Bartlett is, that medical science is to be considered as a branch of natural history, with separate and distinct parts: which parts are to be treated of and studied in themselves. That there are facts, or phenomena, or events, appertaining and belonging to each of these parts, nor can the one class of facts, or any of them be inferred or deduced by *a priori* reasoning, from the facts which are to be found in another, or other parts. That the science of medicine, as all natural science, is a science of observation of facts, or phenomena, or events, with their relationships to other facts, or phenomena, or events; the whole classified and arranged. In other words, that the whole compass and power of the human mind, in the study of natural philosophy, medical science included, is limited to the observation of facts, phenomena, and events, and the classification and arrangement of them. That the mind cannot look beyond what, in a certain sense, it may be said to see. That an hypothesis or theory may lead to experiments, and thus be of some use, but that it can do no more. That the greatest man is the one who has observed (carefully and without prejudice, be it always understood) the largest number of facts, or phenomena, or events, in reference to any science or sciences, and who is the best able to classify and arrange them—to generalize them.

The genius of the minds, which have presided over American schools, and given direction to the young intellects of our new world, is not the genius of labor, of close, careful, untiring observation, of rigid generalization; but the genius of speculation.

Dr. Bartlett is of opinion that the view generally received and acted upon has given rise to a loose and vague philosophy, if we may honor the crude notions commonly taught with that name; that we have jumbled together incongruous things, as one of the old doctors did his drugs, expecting some good to come out of the mixture. Such, indeed, is the fact. So mixed and confused has been the study of medicine, that it is not only difficult to find a well educated physician who can give an intelligible summary of the most important divisions of medical science; but even among teachers we find that they have not so learned medicine. It is a very common complaint among students of medicine that the professors trespass upon the branches of each other; now the true reason for this is, that not one professor in a dozen could complete his course if he did not do so. It is in plain terms, ignorance of their own branch which makes them trespass on that of another; and yet they often say that they have not time to complete their course. The truth is that many of them would not know when it was completed. They have no definite idea of what is included in their department.

Bad as this state of things is, these gentlemen are not altogether to blame for the parts which they act. It is mainly attributable to the sort of philosophising which has prevailed, and to the manner in which medicine has been studied and taught.

Preliminary to the philosophy of medical science, we have in this Essay a condensed view of the principles of physical science. The first proposition is: "All physical science consists in ascertained facts, or phenomena, or events; with their relationships to other facts, or phenomena, or events; the whole classified and arranged: and in nothing else." The author says, "There seems to be a common feeling, that the facts, phenomena, and events, with their relationships, classified and arranged, constitute not the entire science to which they belong, but only the foundation of the science. There is a feeling, that these facts and relationships are to be used as elements, out of which the science is to be built up, or constructed, by what is called inductive reasoning. The feeling implies, and the avowed doctrine asserts, that the science is in this subsequent process of reasoning, and not in the facts themselves, and their relationships." To maintain this general doctrine, and to deny the correctness of the view commonly received is the burden of his labors. What the author says of physical science in the above quotation he insists upon as true respecting medical science, that it consists in ascertained facts, with their relations to other phenomena, classified and arranged. He continues:

"If it is true, even in physical science, as I have endeavored to show, that the doctrine stated in my first proposition, is only partially and imperfectly recognized, it is so to a much greater extent in regard to the same doctrine in its application to medical science; and the remarks already made upon this doctrine may be repeated with still less qualification, and with more emphatic significance, in connexion with my present subject. The fundamental and primary truth, that all medical science consists in the appreciable phenomena of life, with their relationships, classified and arranged, and in nothing else, has never been generally admitted and received. This science, to a vastly greater extent than any other, has always suffered, and still continues to suffer, from the general prevalence of a spurious philosophy, and from vicious or imperfect methods of investigation; and one element in this false philosophy, leading to these mistaken methods, is to be found in the inadequate conception or half-belief of the doctrine above stated. Here, as in physical science with very few exceptions, men, claiming to be disciples of the Baconian philosophy, eloquent in their praises of what they call *inductive reasoning*, and full of earnest declamation against the dangers and the prevalence of false or premature generalizations, and of hypothetical speculation, have failed to see more than half the truth, and have, oftener than otherwise, fallen headlong into the errors which they were so ready to condemn. The feeling has been much more common in medical, than in physical science, that although facts and their relations might indeed, and must constitute the *foundation* of the science, the science still consisted in something more than these facts and relations;—that *upon* these latter the science itself was to be somehow built up, by that magical and creative process of the mind,—that evil genius of medical science,—called indeed, *induction*, but differing, when stripped of its disguises, in no single function or attribute, from that speculation, the place of which it professed, with promises as loud and pompous, as they have proved to be barren and empty, to occupy. The feeling has been, and still is,—as much, almost, since the time of Bacon as before,—that the science is in the *inductive or reasoning process, superadded to the facts and their relations*, more than in these latter themselves. Here, at the commencement of this part of my essay, I wish to enter my protest against this doctrine, in all its forms and modifications. I wish to show, that the science of medicine consists *in the phenomena of life, with their relationships, classified and arranged*,—WHOLLY, ENTIRELY, ABSOLUTELY. I wish to show, that these elements constitute,—not the foundation upon which, nor the materials, merely, with which, the science is to be subsequently constructed, by some recondite and logical process of the reason,—but that they *are* the science, and the whole science, already constructed, and so far completed; and that nothing can be superadded to them, by any act of the mind, which can in any way increase their value, or change their character.

4

The second proposition of our author maintains, that our knowledge of physiology is not deducible from our knowledge of anatomy. He asks the question, "Is there anything in the obvious physical properties of the glands of the human body,—is there any thing in their chemical composition, or in their minute molecular arrangement,—from which even the obscurest, and most shadowy glimpse could have been obtained of the several offices which they are destined to perform? Could the scalpel of the dissector, or the lenses of the optician, or the retort of the analyst, or all combined, have ever revealed to us the power of the liver to secrete bile, of the kidneys to secrete urine, of the mammary glands to secrete milk? The answers to the questions are in the negative—that the most intimate knowledge of the anatomy of an organ does not teach us the function of that organ."

But it does not follow that, because anatomy does not enable us to understand physiology, we can understand the latter without a knowledge of the former. There is, I conceive, some want of fairness in the order in which the subjects are arranged: namely, anatomy, physiology, pathology, therapeutics; although those which precede do not teach us those which follow, yet we cannot understand the subsequent without a knowledge of the antecedent.

We proceed a step further, says Dr. Bartlett. "Pathology is not founded upon physiology. The latter is not the basis of the former. The one does not flow from the other. Our knowledge of the one does not presuppose our knowledge of the other." The chapter from which this extract is made, is taken up chiefly in stating the qualifications of the doctrine just stated. The qualifications are, in our judgment, fundamental, and vast in their consequences. But our author is not of that opinion; and here, before stating the qualifications, we would remark, that Dr. Bartlett, though writing a book in opposition to all medical theory, is, nevertheless, decidedly under the influence of one himself. He is a disciple of the French school, and shews his theoretical bias more by opposing what is called humoralism, than by positively advocating solidism.

The first qualification to which we would call attention, is given by our author as follows: "I have already said that every simple and direct relationship is constant and invariable. Supposing now the physiological actions, relationships of the body, to be fully ascertained, we may safely conclude, independent of positive experience, that a change in those relationships will be followed by a change in the actions themselves, and, and in the results of those actions. Thus after physiology has taught us, as far as it can teach us, the action of the oxygen of the atmosphere upon the blood, we may safely and positively conclude, prior to all experience, and independent of it, that if this action is interrupted, all the subsequent physiological processes, with which it is connected will be also, and necessarily more or less disturbed; and the same thing is true of physiological actions and relationships."

The author thus expresses himself on the same point:

"Finally, so intimate, and complicated, and manifold, are the physiological relationships of the living economy; so closely is each part connected with the rest; so readily and powerfully do these parts act and react upon each other; so complete, in many instances, is the union and the coöperation of the mechanical, the chemical, and the vital processes, that, independent of actual experience, we might safely conclude, that an injury inflicted upon one part of the body might often affect, more or less seriously, many other parts; and that a disturbance, or suspension. of any one of the three great processes might, in many cases at any rate, disturb, or suspend, the other two. Having ascertained, by observation, the complicated physiology of the circulation and of respiration; having ascertained the existence of various mechanical, chemical, and vital actions, and their necessary coöperation in order to produce a certain result, consisting in the circulation, the oxygenation, and the decarbonization of the blood; it would follow, as a matter of course, and independent of positive experience, that a disturbance, or suspension of one of these associated actions, should disturb, or suspend, the others. Inasmuch as the integrity of the mechanical contrivances for the repeated exposure of the blood to the influence of the atmospheric oxygen is necessary, to secure this exposure, and inasmuch as this exposure is necessary, in order that the oxygenation, and decarbonization of the blood should be effected; and inasmuch as this change in the blood is essential in order to prepare it for answering its purposes in the vital processes of the body, it follows, necessarily, that any disturbance, or imperfection, in the first mechanical process, will be followed by corresponding disturbances and imperfections in the subsequent and associated chemical and vital processes. In the same way, also, having ascertained that certain substances are eliminated from the body by the physiological actions of the liver, and the kidneys, we might justly come to the conclusion, without waiting for the positive teachings of experience, that the retention of these substances within the system would be followed by unfavorable results."

We would ask, do not these qualifications cover the entire ground of functional disorder? Do they not apply with great force to the causes and nature of miasmatic fevers? Do they not apply to scrofula, since Baudelocque has so conclusively shewn that it originates in the breathing for a length of time of bad air, or air intermixed with carbonic acid or other gases? But the author takes a very different view of them. In justice to his profound and extensive knowledge of his subject he was compelled to make them. But will not every reader be astonished to hear him say—"they are more nominal than real. When closely examined and analysed they reduce themselves within very insignificant dimensions."

This multiplication of his own qualifications, which we maintain are fundamental, philosophical, and true, proves our author to be himself a theorist, and that he attaches undue importance to lesions of structure.

If it is granted as a fact, that from a particular cause, miasm for instance, the blood has been altered in its composition, we can as readily comprehend how certain organs must become disordered, as we could in the first instance understand how the blood became changed. It is not a specultion to say that the liver will be implicated under the continued operation of such a cause; and that local congestions, as of the head, lungs, and abdominal viscera, may be anticipated.

The departure from the healthy, natural action of vital organs is all that we have to guide us in many cases of the worst form of disease. We may know that a particular lesion exists, but it profits us nothing. We cannot reach the local affection until we remove the difficulty in the way of vital functions; and the means by which this is to be accomplished have no reference to the lesion whatever.

That our author belongs to the class of solidists, we have only to refer to his chapters on intermittent, remittent, and yellow fevers, in his work on typhus and typhoid fever, to be convinced. In his remarks on these fevers we have clear evidence of his leaning to this particular school. Else why should he refer to the writings of men who have seen scarcely anything of the diseases under consideration, when there are so many volumes abounding in facts observed by men who lived for years within the tropics, or near them; by men of the most thorough medical education, and remarkable for the patience and carefulness of their observations; men who tested their theories by years of laborious and successful practice? Why should he refer to Trousseau, Louis, Gerhard, and Stewardson, and pass by Bancroft, Pringle, Lind, Cleghorn, Mackintosh, and James Johnson, not to mention others of equal celebrity? Simply, as we conceive, because the gentlemen to whom he is pleased to refer, with Louis at their head, advocate the system which he adopts; and because they have devoted more time to the examination of anatomical lesions.

It would be difficult to find a more remarkable instance, than is furnished by our learned and ingenious author, of the impossibility of writing on medicine without becoming the advocate of some theory or system. Evidently his is a mind thoroughly imbued with the true inductive philosophy; his conceptions of what constitutes science are eminently just, and yet while repudiating all hypothesis he gives indubitable proof of being under the dominion of a mere hypothesis. His work might be called an essay on the architecture of medicine. It places in bold relief the different columns and pillars; he has wrought out each part in exact and due proportions; they are finished in a masterly manner. But the architect is unwilling that the edifice should be constructed. He must have each part stand separate and alone; and although he sees that the parts are made to fit into each other, yet he declares that this is only in appearance, and not a reality—that they are in fact isolated, and so they must stand.

We agree with Dr. Bartlett, that anatomy, physiology, pathology, and therapeutics are separate and distinct; and that to suppose that a knowledge of one will give us a knowledge of the other is a great mistake,—one which cannot be corrected too soon. We believe that a vast amount of false philosophising has prevailed and still prevails on these subjects; we believe that the geologist who first discovered and made known the arrangement of the strata of the earth into primary, secondary, and tertiary rocks, with their several subdivisions, scarcely did more for the student of geology than our author has done in this work for the student of medicine; and had he been free from the bias of the French school he would have made his essay far more valuable than it is. But he had adopted in theory, and acted upon in practice, M. Louis's method, long before he wrote a page of his work; and we cannot resist the belief that his object was not more to correct errors which really exist, than to lay the foundation for M. Louis's system.

If pathology consists in the facts, or phenomena, or events of disordered action, with the relationships of these facts classified and arranged, as in fact it does, may we not say that there are relationships existing between these facts, and others observed in the domain of physiology? Should not the physician at the bed-side of a patient endeavor to ascertain every fact, whether it be found in one or the other departments of the science, and then consider its relationships? Is it contrary to sound philosophy to take a comprehensive view of all together? We think not, and we are obliged therefore to deprecate this unreasonable disposition to build up a wall of separation between the different branches of the science.

Man is a living being, and in the same body we find, so far at least as the solid organism is concerned, diseased and sound parts. We shall never be able to draw the line of separation between the sound and the unsound as we can between a stratum of primary and of secondary rock; nor can we weigh the quantum of health and disease as we can the chemical elements of a block of marble.

Dr. Bartlett thinks that diseases may be classified and arranged as plants are, according to the natural system of botany. We greatly doubt this. We incline too much to the humoral doctrine to allow the practicability of any such thing.

Our author is unable to believe that our facts are sufficient to prove that malaria is the cause of intermittent, remittent, and yellow fevers. Now it strikes us that the reason for this remarkable scepticism is to be found in the fact, that almost all who hold to the miasmatic origin of the fevers in question, believe that it enters the circulation through the lungs along with the air we breathe. This view involves so many humoral consequences, which are inevitable, that to avoid them, Dr. Bartlett boldly denies the first position—that miasmata are the cause of these fevers.

Dr. Bartlett seems to think that because the successful practice of medicine has not kept pace with the improvements and discoveries which have been made in some of the branches of the science, we have corroborative proof of his position, that a knowledge of the one branch does not teach us a knowledge of the other. He refers to Mr. Jefferson's opinion concerning the practice of medicine before and after the discovery of Harvey. If medicine was practiced as successfully before the discovery of the circulation of the blood as it has been since, the fact is in opposition to the doctrine of our author. We believe that many men have been more distinguished as successful practitioners than is M. Louis at this day. Indeed Dr. Bartlett admits himself that English physicians are more successful as practitioners than the French.

Before pathological anatomy came to be thought so very necessary a part of medical education, and the doctrine of lesions became the order of the day, physicians made out their diagnosis more by a comparison of disordered with healthy actions, than in any other manner. They did not go farther perhaps than Dr. Bartlett allows us to go in his qualifications to his general doctrine, but that was far enough; and upon such principles they practised successfully; more so, perhaps, than Louis, or Chomel. To shew how uncertain are pathological appearances after death we may quote M. Louis's opinion of Broussais. He holds that Broussais, with all his knowledge and extensive observation, was not qualified to pronounce upon the cause of death after a post mortem examination. Broussais was a "theorist;" and so may be Louis; and so may be our author.

When Dr. Bartlett says that therapeutices is not founded upon phathology, he merely intends to say, as we conceive, that our knowledge of a particular lesion does not enable us, *a priori*, to determine upon the remedy. We do not understand him to mean, that after a remedy has been discovered, there may not appear to be a relationship between the disease and the remedy. We will give an illustration.

When Champollion first directed his attention to the Egyptian hieroglyphics he could not conceive of their import; but after the Rosetta stone gave him a clue, he went on gradually to read, until after a time, he was prepared to relate to the world the mysteries of the pyramids. So in the science of medicine. If our remedies act in accordance with the laws of the animal economy at all, there must be a relationship between the diseased part and the remedy; accident may discover it at first; but after it has been discovered, it is not unreasonable to suppose that the mind may perceive that relationship. When we shall come to understand diseases more clearly, when we shall have more carefully investigated the action of remedies, and learned better to discriminate between the physiological and therapeutical action of a medicine, we shall be able to perceive these relationships more clearly, and no doubt discover a greater number of them.

We have been reluctant to differ with our excellent and distinguished author on these points, but notwithstanding the errors into which we are constrained to believe he has fallen, our estimate of his work is such, that we cordially recommend it to every young physician as a most useful aid in the prosecution of medical study. We believe the award of posterity concerning it will be favorable; and for ourselves, we are agreed that the medical philosophy of our century shall be judged of by it. J. C. D.

Lexington, April 21st, 1845.

